# Controlled human infection study underpins efficacy of the tetravalent live-attenuated dengue vaccine TV005

**DOI:** 10.1172/JCI177610

**Published:** 2024-02-01

**Authors:** Annelies Wilder-Smith

**Affiliations:** Disease Control, London School of Hygiene and Tropical Medicine, London, United Kingdom.

## Abstract

Dengue fever, caused by four distinct serotypes of the dengue virus (DENV1–4), poses a public health concern for much of the world. The NIH’s Laboratory of Infectious Diseases at the National Institute of Allergy and Infectious Diseases (NIAID) has developed a series of single-dose, live-attenuated tetravalent DENV vaccines, including TV005. However, phase III trials require a lengthy three-to-five year follow-up. In contrast, controlled human infection models (CHIMs) offer a faster means to assess vaccine efficacy for any of the four serotypes. In this issue of the *JCI*, Pierce, Durbin, and colleagues conducted a CHIM study with attenuated DENV2 and DENV3 challenge viruses in individuals six months after vaccination with TV005. The TV005 vaccine was well tolerated and effectively protected all vaccinated individuals from viremia and rash during challenges with DENV2 or DENV3. Notably, vaccine recipients also showed serotype-specific efficacy. While long-term studies are still needed, these findings represent an important step in providing protection against dengue virus.

## Vaccines against dengue virus

Dengue fever, a widespread arboviral infection caused by four distinct serotypes of the dengue virus (DENV1–4), continues to be a substantial public health concern in numerous tropical and subtropical areas. Its incidence and geographical spread are expected to rise further (1). The risk of more severe disease outcomes depends on the number of infections: a second infection with a different serotype is associated with an enhanced risk for progression to severe dengue including vascular leakage syndrome (2). The NIH’s Laboratory of Infectious Diseases at the National Institute of Allergy and Infectious Diseases (NIAID) has developed a series of single-dose, live-attenuated tetravalent DENV vaccines. Among these, TV003 and TV005 are leading candidates, demonstrating the most effective balance of immunogenicity and safety and a balanced immune response against all four serotypes (3, 4). Both TV003 and TV005 consist of identical monovalent components, differing only in the serotype 2 dosage — TV005 contains a ten-fold higher dose, at 10^4^ PFU, to address the overly attenuated serotype 2 component.

Comprehensive phase I and II trials have been conducted for TV003 and TV005. In Brazil, the Instituto Butantan is nearing the completion of a five-year phase III clinical efficacy trial using a single dose of TV003. In Bangladesh, a phase II trial with three years of follow-up showed that a single dose of TV005 was well tolerated and immunogenic for all four serotypes in a range of ages, from young children to adults, including individuals with no previous dengue exposure (5). Conducting phase III efficacy trials for live-attenuated dengue vaccines is complex and lengthy, partly because of WHO guidelines requiring baseline blood samples from participants for efficacy stratification by serostatus and the need for a three-to-five year follow-up to identify potential safety risks, which may occur about three years after vaccination (6).

In contrast, controlled human infection models (CHIMs) offer a faster means to assess vaccine efficacy for any of the four serotypes, once a suitable challenge virus is established. CHIMs involve a controlled inoculum, a precise infection timeline, and thorough clinical monitoring. Importantly, data from CHIM experiments can be used to extrapolate vaccine efficacy for each serotype independently. However, to date, challenge viruses for dengue fever have only been developed for DENV2 and -3; a challenge virus for DENV4 is currently being developed.

For the evaluation of TV003, a CHIM was used to confirm protection against a DENV2 challenge virus six months after vaccination and showed complete protection in volunteers (7). A DENV3 challenge virus was only recently developed. The integration of additional serotypes and live-attenuated tetravalent vaccines are needed to optimize vaccine development.

## The TV005 vaccine protects against viremia and rash

**Figure 1:**
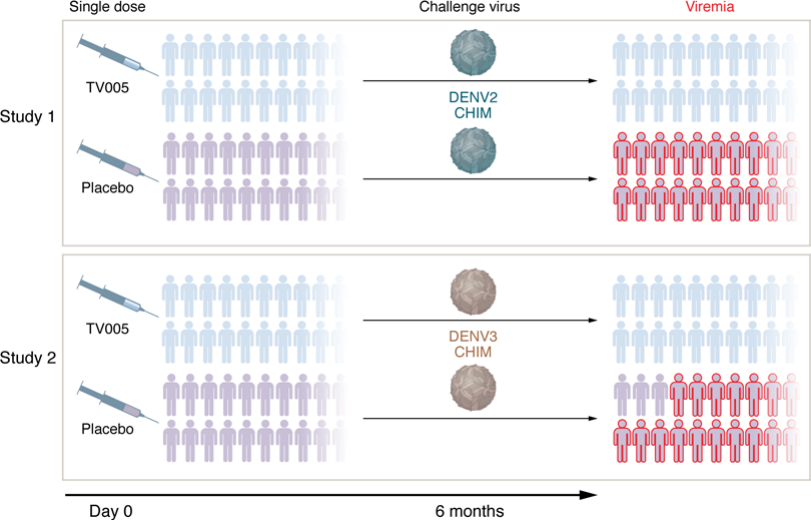
TV005 protects participants in controlled human infection studies. Six months following a single dose of TV005 or placebo, participants were challenged with attenuated dengue DENV2 or DENV3 virus. The TV005 vaccine effectively protected vaccinated individuals from viremia and rash in both challenges. All participants (100%) who received placebo and the DENV2 virus developed viremia, and 85% of participants challenged with the DENV3 virus developed viremia.

In this issue of the *JCI*, Pierce, Durbin, and colleagues explored a controlled human infection study conducted with attenuated DENV2 and DENV3 challenge viruses in individuals six months after vaccination with TV005 (8). The primary goal was to ascertain the efficacy of TV005, gauged by viremia levels. Secondary objectives included the evaluation of safety and analysis of immunological and virological responses. The sample size of approximately 42 individuals for both randomized, controlled trials aligns with other CHIM studies (8) (Figure 1).

The TV005 vaccine effectively protected all vaccinated individuals from viremia and rash during challenges with DENV2 or DENV3. In contrast, 100% of individuals in the unvaccinated control group developed DENV2 viremia after a DENV2 challenge, and 85% developed DENV3 viremia following a DENV3 challenge. Most vaccinated participants developed neutralizing antibodies against multiple serotypes within 90 days of receiving TV005. Serological responses to three serotypes were observed in 100% of vaccine recipients (known as a trivalent response), and 72% of recipients showed responses to all four serotypes (known as a tetravalent response). DENV2 viremia was generally higher and lasted longer than DENV3 challenge viremia. The vaccine was generally well tolerated and safe. The majority of vaccine recipients experienced a post-vaccination short-lived mild, almost asymptomatic, rash, consistent with previous findings (8, 5).

It should be noted that the challenge strains were attenuated by the deletion of nucleotides in the untranslated region of the viral genome. Hence, no extrapolations can be drawn with regard to efficacy against severe dengue. However, one could assume that preventing infection would also translate into preventing severe dengue. Furthermore, study participants were dengue naive. Since second infections with different DENV strains manifest as more serious disease, additional studies and approaches are needed to confirm vaccine protection against wild-type dengue, both in dengue-naive and dengue-exposed persons. Furthermore, the findings do not encompass long-term safety, as the challenge occurred only six months after vaccination. Considering the experience with the first licensed tetravalent live-attenuated dengue vaccine CYD-TDV (Dengvaxia), the safety signal was observed approximately two to three years after vaccination in dengue-naive persons (9), hence, this CHIM cannot exclude long-term safety issues beyond six months. Until immune correlates for protection and risk have been established, continued real-world clinical trials that include long-term observation remain essential. Although CHIMs cannot replace phase III trials, the CHIM results from Pierce, Durbin, and co-authors (8) add valuable data on serotype-specific efficacy and safety and provide an impetus to further advance the TV005 vaccine.

The NIAID has licensed TV003 and TV005 as single-dose vaccines to various manufacturers, including those in low-to-middle income countries, ensuring future access to TV003 and TV005 once regulatory approvals are granted. The next step in the regulatory pathway is the release of the five-year phase III trial results for TV003.

Meanwhile, the WHO has endorsed the second licensed dengue vaccine, TAK-003 (Qdenga), developed by Takeda for use in high dengue burden settings (10).

## Version 1 02/01/2024

Electronic publication
